# Steering without navigation equipment: the lamentable state of Australian health policy reform

**DOI:** 10.1186/1743-8462-6-27

**Published:** 2009-11-30

**Authors:** Jeff RJ Richardson

**Affiliations:** 1 Centre for Health Economics, Faculty of Business and Economics, Monash University, Clayton Victoria 3800, Australia

## Abstract

**Background:**

Commentary on health policy reform in Australia often commences with an unstated logical error: Australians' health is good, therefore the Australian Health System is good. This possibly explains the disconnect between the options discussed, the areas needing reform and the generally self-congratulatory tone of the discussion: a good system needs (relatively) minor improvement.

**Results:**

This paper comments on some issues of particular concern to Australian health policy makers and some areas needing urgent reform. The two sets of issues do not overlap. It is suggested that there are two fundamental reasons for this. The first is the failure to develop governance structures which promote the identification and resolution of problems according to their importance. The second and related failure is the failure to equip the health services industry with satisfactory navigation equipment - independent research capacity, independent reporting and evaluation - on a scale commensurate with the needs of the country's largest industry. These two failures together deprive the health system - as a system - of the chief driver of progress in every successful industry in the 20th Century.

**Conclusion:**

Concluding comment is made on the National Health and Hospitals Reform Commission (NHHRC). This continued the tradition of largely evidence free argument and decision making. It failed to identify and properly analyse major system failures, the reasons for them and the form of governance which would maximise the likelihood of future error leaning. The NHHRC itself failed to error learn from past policy failures, a key lesson from which is that a major - and possibly the major - obstacle to reform, is government itself. The Commission virtually ignored the issue of governance. The endorsement of a monopolised system, driven by benevolent managers will miss the major lesson of history which is illustrated by Australia's own failures.

## Background

Concerns which have dominated national debate and government attention have commonly reflected vested interests and ideologies rather than the evidence-based magnitude of problems. The different interest groups include, as they have always done, the medical profession, private health insurance (PHI), private hospitals, increasingly, the pharmaceutical industry, the public health lobby and 'government economic rationalists'.

One ideology concerns the unsubstantiated superiority of varying levels of private ownership, control and financing in the health sector. Another ideological belief is that health spending should be dedicated only to health maximisation (ignoring some notions of freedom and fairness). Then there is the ideology of many government departments - especially those heavily influenced by economists - that small government is an end in itself and that minimum resource cost per unit of measured output is always desirable. In the health sector this latter ideology does not reflect population values [1].

In contrast with these views, there is a strong argument for public spending to be based upon evidence, including evidence relating to public values. This, of course, requires information, but currently much of the information needed to achieve this apparently obvious goal does not exist, that is, the health system is being steered without satisfactory navigation equipment.

In the present paper I initially comment upon three of the prominent issues in the health debate, each of which is associated with a powerful constituency namely, private health insurance (PHI), ageing and hospital queues. Privatisation could be added as a fourth. The theme of this brief discussion is that the quality of the analysis has been poor to the extent that it borders, at times, upon disinformation. This raises the question of how this could occur. In the following sections I outline evidence of more significant system failure - the regulation and diffusion of technology, the fairness of the system and the quality of care. Relative to their importance these issues have been largely ignored in the health debate and attracted, at best, a lethargic policy response. This again raises the question of how this could occur.

In the remainder of the article it is argued that the answer to these questions is, in large part, that the health system has poor governance and has failed to invest adequately in research and experimentation. This is symptomatic of a more fundamental problem, namely the near monopolisation of each part of the system by conservative and defensive government agencies and the belief that deficiencies may be corrected by (occasional) one-off tinkering with the system rather than by the creation of a system based upon the production and diffusion of evidence, health services research commensurate with size and importance of the health sector and upon error learning rather than error suppression. Some principles for achieving this are discussed.

## Issues of Exaggerated Importance

### Private Health Insurance (PHI)

To put PHI in perspective there are two dominating facts. First, the imperfect evidence available suggests that many Australians wish to have PHI. National statistics on the redistributive mechanisms in Australia and the resultant levels of poverty suggest that Australia is one of the least egalitarian and the least generous nations in the developed world (see Table 1 and Additional File 1). Results from the Monash Health and Ethics Survey [2] reveal overwhelming support for the proposition that people should be allowed to spend additional monies on their own health. Many Australians like to jump queues and support multi-tier access to health services.

**Table 1:** How Australia compares

The rank order of Australia compared with 18 other OECD* countries: selected statistics				
	**Year**	**Rank**	**No. of countries**	**Australia's ranking**

% population in absolute poverty	1995	10 = highest	10	10

Social security transfer (% GDP)	1990-1999	17 = lowest	17	17

% elderly in poverty	Late 1990s	16 = highest	16	16

Income of elderly (above 65). ÷ Income 18-64	Mid 1990s	16 = lowest	16	16

Income at 90^th^/income at 10^th ^percentile	Late 1990s	17 = highest inequality	17	13

% population with income below 50% of median income	Late 1990s	16 = highest	16	15

% children in poverty				
- single mother - two parents	mid 1990s	15 = highest 15 = highest	15 15	13 13

Official Aid/GDP	2000	18 = lowest	18	15

Total tax/GDP (%)	2000	1 = highest tax	18	15

* All OECD countries with a population above 3 million

Source: [42]

The second dominating fact is that, in terms of its importance for the sustainability of the health sector, PHI is quantitatively trivial. In 2007/08 it raised $7.86 billion of the total national health expenditure of $103.56 billion or about 7.6 percent. Even in the hospital sector where its funds are concentrated, it raised only 11 percent of revenues [3]. The highly publicised statistic that PHI underpins private hospitals which, in turn, carry out over 50 percent of elective surgery is one of the many examples of disinformation, or perhaps more accurately, 'spin', on the statistics. Health funds and private hospitals are the landlords providing beds and equipment. The Government, not PHI, provides the overwhelming proportion of the insurance against the medical costs in these hospitals.

It would be feasible (as distinct from necessarily desirable), for private hospitals to be nationalised overnight with 57 percent of their contribution to hospitals being met from the government subsidy to premiums and the remaining revenues raised by PHI met by an approximate 1.1 percent increase in taxation. This would not be an economic cost to the nation but a redistribution from non contributors to PHI to those who are currently members. This course of action is neither advocated nor criticised here. The key point is that, financially, PHI is an inessential part of the health sector. The Australian health scheme cannot be held to ransom by the self evidently false claim that it depends upon PHI.

Finally, it is difficult to even measure the underlying support for PHI as the measures taken to support it have distorted the relationship between preferences for PHI and purchasing decisions. Legislation in the last decade has driven Australians into PHI with policies which deserve to be enshrined in the Guinness Book of Records - as the most bizarre micro-economic policy in a developed country in the last half century.

The levy on the wealthy who fail to purchase PHI has the same economic logic as promoting the Australian automobile industry with a punitive tax surcharge on the income of wealthy Australians who fail to buy an Australian car. Lifetime tables are even more perverse. Insurance, which is usually envisaged as a mechanism for reducing risks has, by legislation, been forced to increase risk. The uncertainty associated with illness over the next 20-30 years is clearly much greater than the uncertainty associated with the next 2 to 3 years. Those making a decision with a respect to the purchase of insurance are now faced with greater anxiety and fear because of the longer decision period, and fear drives people to insurance. An imperfect analogy would be a policy to increase the uptake of fire insurance for houses by randomly burning down houses, increasing people's fear of being a victim and thereby inducing them to take out fire insurance.

Under these circumstances it is difficult to determine the underlying demand for (legitimately subsidised) PHI, but survey results cited above indicate a rejection of the notion that individuals should not be permitted to spend more of their own income to receive better access to better services.

Perversely, the subsidy for those taking PHI which is the most criticised policy is the most justifiable in terms of social and normal economic policy. It is justified, however, not as a device to increase membership per se, or health care revenue but in terms of (one notion of) equity to those who are paying more for their health care. This argument depends upon PHI having no adverse effects upon others by, for example, diverting a disproportionate number of doctors from public hospitals). It is untrue, as some advocates from the public health lobby have argued that the subsidy is bad economic policy or that the resources are wasted. Rather, a subsidy is the normal device for promoting a social policy and resources are transferred not wasted. But throughout the health care debate issues of social values are routinely misrepresented as issues of economic (in) efficiency.

### Ageing

The belief that there is a looming crisis because of the ageing of the population and booming demands of those who thoughtlessly chose to be born after 1945 appears to be a function of bad arithmetic. It reflects the well known error of reasoning in percentages and disregarding absolute values. Exaggerating somewhat, the percentage of centurions may well increase by 600 percent in the next 20 years, a frightening prospect until translated into an absolute increase from 100 to 700, a figure too low to have a detectable impact on health expenditures.

**Figure 1:**
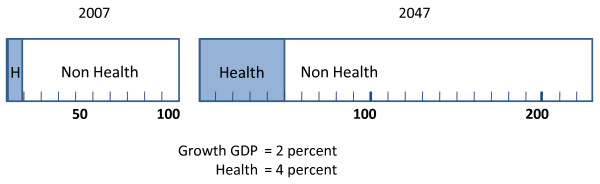
**The magnitude of health and GDP 2009-2049**.

Importantly, and unremarked in reports, a much higher rate of taxation driven by a much higher growth rate of health expenditures is consistent with a rising material standard of living. This may be verified by anyone capable of calculating compound growth rates. Figure 1 illustrates the effect of health expenditures rising at twice the rate per annum as GDP. The bar diagram on the left depicts the GDP visually as an index of 100 and the proportion of this devoted to health services. The bar on the right illustrates the effect upon resource use and availability in 40 years time if health services grow at 4 percent per annum and GDP at half this rate. GDP would rise by a factor of 2.208. Health expenditures would increase from 10 to 22 percent of this. However, resources left for other expenditures would increase by 90 percent. This is the simple arithmetic result of a larger magnitude (GDP) growing more in absolute terms than a lesser magnitude (health) despite rising less in percentage terms. The key message is relatively insensitive to the numbers used in the example; viz, it is almost inconceivable that rising health expenditures could significantly contribute to a reduction in the GDP/capita.

Even the use of GDP/capita as a benchmark is largely a product of history and intellectual inertia as the available evidence shows no relationship between GDP/capita and individual wellbeing [4,5]. Policy, however, appears to be driven by a fear of changes in the percentage composition of the GDP. But there is no economic or social reason why the composition should not change, especially if it increases wellbeing. Historically, the economy has been flexible. The agricultural sector, for example has 'imploded' in percentage terms without adverse effects. The service sector, including health, has expanded to our advantage (and possibly to the benefit of the environment).

**Figure 2:**
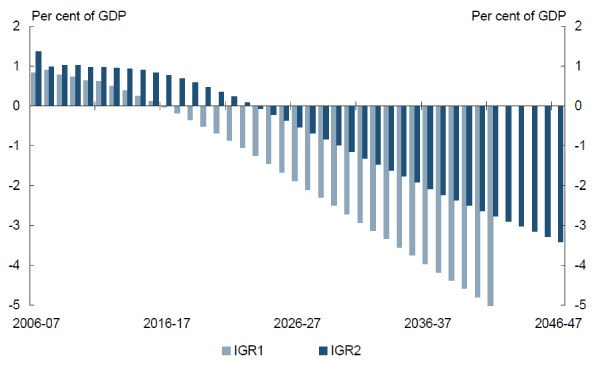
**Disinformation: Intergenerational Report 2007, budget deficit as % of GDP**. Source: [6].

The Intergenerational Reports and Productivity Commission have carried out analyses in which the loss of perspective results in serious disinformation [6,7]. Figure 2, taken from the beginning of the 2007 Intergenerational Report and reproduced widely, encapsulates one of the widely cited headline 'messages' from the report. This is that the budget deficit as a percent of GDP will rise alarmingly to the year 2046-47 and in a way that is directly attributable to increased health expenditures. The increased government health expenditures are shown to exactly match the increased deficit. However the report does not make clear - at least in its headlines - that its projections are based upon the assumption that past trends will continue inflexibly and that taxation is pegged at the level necessary to support service use in 2007. The Productivity Commission reaches similar headline conclusions *albeit *with qualifications deep in the body of the report. The National Health and Hospitals Reform Commission has clearly accepted this message and warns that health expenditures could so dominate State budgets that there would be no money left for roads or education [8].

In fact these results are entirely attributable to a set of assumptions which are tenuous at best and wrong at worst. In the light of recent macro-economic experience economic prediction to the year 2046-47 should be undertaken only in satire. However four points should be obvious:

1. The economic burden of health spending depends upon the rate of growth of GDP.

2. Ageing per se in the absence of new health technology but with economic growth would have a minimal effect on the burden of health expenditures. Even with zero GDP growth and no other changes age driven expenditure would rise between 1997 and 2051 from 7.5 to 11 percent of GDP [9].

3. Extrapolations are based upon assumptions. In the present case the effect of ageing has been obtained by adding technology driven trend health expenditures to the ageing effect, not by an ageing effect per se. However, the impact of technology over a longer period of time is far more difficult to predict than relatively inexorable changes in the medium term population structure.

4. Technologies may be cost enhancing (as at present) or cost reducing (as with the introduction of antibiotics and possibly products arising from the present biotech revolution). Unlike population, technology can be regulated in such a way that increased expenditures should be welcomed if the benefits exceed the costs.

**Figure 3:**
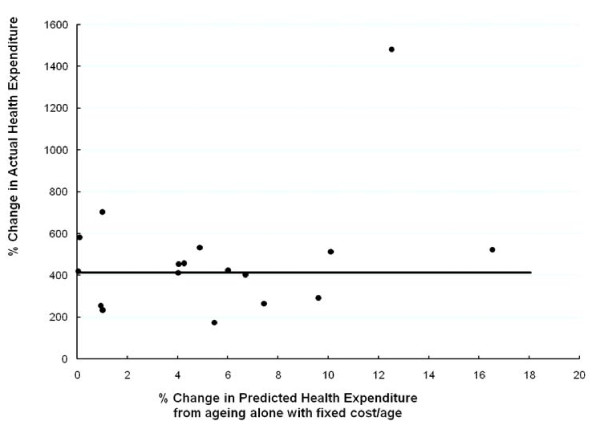
**Change in health expenditures compared with the change in age/sex predicted expenditures, 1960-95 (21 OCED countries)**. Source: [9].

To date the varying rates of population growth across Europe have been uncorrelated with expenditure growth. Figure 3 plots the actual growth for each country on the vertical axis against the rate which would have resulted from ageing in the absence of any other influence and specifically technological change. The result indicates that ageing effects have been absorbed into general expenditure growth. Historically, the inextricable link between ageing and health expenditures as a percent of GDP implied by government reports and the press is simply disinformation.

The final assumption underlying these defective analyses is that taxation will remain fixed as a percentage of GDP. Tax rates in Australia are amongst the lowest in the Western World (Table 1) and could rise by 50 to 60 percent before reaching the levels of countries whose rates of economic growth and standards of living have been unaffected by their higher tax rates.

Policy analysis may have been driven by bad arithmetic. A less benign interpretation is that economic advisors are presenting data to achieve a covert objective suggested by bad neoclassical economic theory. There is an arcane belief in this discipline that additional taxation carries an 'excess burden' for human wellbeing. I have described it as arcane because there is no empirical evidence for this belief and the logic behind it is wrong. It assumes that with less tax people work harder and are thereby better off (or else why would they have worked harder?) The underlying assumption is that the undistorted balance between work, leisure and all other elements relevant for human wellbeing is in an optimal state before the distortion of marginal taxation. The assumptions behind this evidence-free belief are so absurd they will not be repeated here.

It is worth noting, however, that while the excess burden 'doctrine' teaches that each dollar of tax has a disproportionate cost, economic theory has not suggested a way of demonstrating the excess or deficit value of most of the Government activity funded from these taxes. It is implicitly assumed in the GDP accounts that health, education, law and defence contribute to wellbeing an amount equal to their dollar cost (and therefore less than the 'cost' of taxation). Transfer payments to the disadvantaged also do not generate net benefits but only redistribute them as, according to orthodox theory, we cannot compare the utility benefits of one person with another. Transfers may, however, distort work incentives and, for the same arcane reason as above this is harmful. Consequently, and without evidence, tax based transfers become a net cost. The benefits must be treated on a dollar for dollar basis; the taxes inflict more than a dollar for dollar cost when taken and given. This may help explain the clearly prejudicial attitude of many economists towards taxation. It does not justify their insinuating policy by stealth.

### Hospital Queues

Queues or surpluses are difficult to avoid in the context of a free service. Despite the understandable concern and publicity, however, these should not be an important issue in the long term. Three brief comments are relevant here.

1. It is illustrative of one of the themes of this paper that policy options which are known, in principle, cannot be put into action with any confidence because of the inadequate research into hospital and medical behaviour, the inadequacy of information systems, the lack of experimentation and the avoidance of system reforms designed to optimise the use of acute hospitals.

A partial solution to the problem, emphasised by government, is to increase hospital efficiency. Hospital information systems, financial incentives and other internal reforms have a role to play of course. Hospital pressures can also be reduced by rational step down and step up policies, from expanded primary health care and other low intensity facilities in conjunction with appropriate admission and discharge policies. But system reforms and poor governance structures have inhibited experimentation and discouraged development of these.

In the long term, however, queuing has little or nothing to do with hospital efficiency. It is the outcome of the balance between supply and demand. There is, in principle, near universal agreement that realised demand should reflect acceptable access to Medicare and admission for prescribed needs. Ultimately queuing can only be altered by changing one of these variables: that is, queuing must ultimately be regulated by changing effective access or supply and the latter can be set at a level which will create or remove unacceptable queues with or without hospital and system efficiency. There has been no long term research, to the author's knowledge, to determine the level of acceptable supply and access. Policy rhetoric emphasises deflectionary, *albeit*, important issues of efficiency.

2. However Australian Governments appear to be primarily concerned with eliminating monetary and not true economic inefficiency. Extremely accurate budgetary data are collected. Virtually no data exist relating to the inefficiency inflicted upon patients in the form of suffering and disruption to their lives (as noted above). Similarly there has been minimal real interest in equity, evidenced by the fact that the public has not been properly consulted. Recent research at the CHE [10] clearly demonstrates that, in the health sector at least, there is an overwhelming concern with fairness and sharing and relatively little interest in monetary efficiency, even when this is expressed in terms of maximising the number of life years gained from a budget. Indeed, the research indicates that the general community would sacrifice 1/3 of life years which could be allocated to people in order to achieve equitable sharing. This strongly suggests that the policy of minimising expenditure - extracting an annual productivity bonus and 'letting health departments cope' - strongly conflicts with the preferences of the population. 'Inefficiency' in the form of greater tax financed expenditure would clearly be preferred to the inequity of non treatment. In view of the evidence that there is no change in subjective wellbeing as GDP rises, [4,11] policies involving increased taxes and spending in areas of demonstrated public concern should be welcomed.

3. The past government's most publicised solution to queuing - incentives for individuals to purchase PHI and private hospital care to 'take pressure off public hospitals' was, at best, evidence free avoidance of the problem, but more consistent with a policy of appeasing the interests associate with PHI. It has been known for at least a decade that queuing has been associated with supply side constraints, not uncontrollable demand. This is exacerbated, not ameliorated, by the transfer of medical staff to private hospital facilities where they are likely to provide more, not less, intensive servicing for the same condition promoted by fee for service and PHI [12-14]. The ability of government to base its policy for so long upon public disinformation attests the lamentable state of public and journalistic understanding of even the simplest relationships in the health sector and the relevant magnitudes.

### Relatively Neglected Issues

#### Technologies

New technology has been the great driver of human welfare generally and new technologies have dominated and will continue to dominate both the costs and the benefits of health services. While face to face consultations per capita fell 6 percent in the 12 years to 2007/08 diagnostic and procedural services rose by 62 and 32 percent per person respectively and the overnight separation rate per 1,000 for acute services rose to a level 27 percent above the rate in the USA, 20 percent above the rate in the older UK population and 66.7 percent above the rate in the comparable Canadian population [15]. Lack of research prevents us from judging whether these large magnitude differences indicate a strength or a weakness in the Australian system. Despite this, lamentably small attention is given to health technologies at the level of health service diffusion and delivery. While Australia pioneered the economic evaluation of drugs, and the use of products and devices, and technologies must be approved by the TGA, the overall research effort in this area is dispersed, uncoordinated and reactive and appears to have little error learning capacity. There is insufficient national capacity to evaluate old therapies retrospectively or to monitor outcomes. The data for this is collected, but is not used.

Long term, there is an urgent need to install the capacity to proactively seek new technologies and to ensure (or block) their diffusion and use as quickly as possible. To fail to do so is to reduce population health. But the issue has never been on the political radar.

#### Equity

Medibank and Medicare were established to achieve equitable access to hospital and medical services. They achieved this in one respect only, namely the extension of financial insurance to the 15 percent of the population who had not purchased it privately prior to Medibank. Apart from this small (*albeit *important) improvement the achievement of equity has remained very largely at the rhetorical level. Using the first universal database from Medibank Richardson and Deeble found that the use of both GP's and Specialists in Sydney in 1976 were both (coincidentally) 4.6 times greater than in Darwin after adjusting for age and sex. Discrepancies between statistical divisions were much greater [16]. Studies by the author 25 years later found similar discrepancies in the use of procedures across Victoria and huge differences in the use of new technologies in the public and private sectors [17,18].

The response to the obvious inequities between urban and rural areas in Australia may best be described as 'dab policies'. There has been little serious attempt to equalise access. The issue, however, has not been near the top of the public agenda and possibly because information about inequities is not routinely documented and distributed throughout the community. Indeed, from the author's experience, it is possible that the non provision of information which has reinforced this complacency may have been promoted by public authorities (see Additional file 2).

Another dimension of inequity relates to the adequacy of insurance coverage by type of medical service. As shown in Table 2, insurance coverage by Medicare is highly erratic reflecting the historical influence of the professional groups benefiting from the insurance (default free funding). Hospital services, the most expensive form of care, are almost fully insured and consequently, cheapest to use. Medicines are very poorly insured despite the fact that the longevity of Australians is probably attributable, in large part to the widespread availability and use of anti-hypertensive drugs. But for many people at risk of CVD these are 'outside Medicare' - there is no subsidy. For example, with an average price to patients of $15.11 per script in 2009 [19] people without a health care card would pay the full price of the antihypertensive drug Atenoll, the sixth largest volume PBS drug dispensed and those with a card face a 30 percent copayment which, evidence suggests, will impact disproportionately upon low individual incomes as there is no immediate symptom relief [20]. Possibly the most cost effective therapy in the health system is effectively excluded from Medicare for the majority of the population and the disadvantaged pay large copayments. Of all cardiovascular drugs on the PBS 15.8 percent receive no subsidy and an unknown number a very low subsidy [19]. There is no stated rationale for discriminating against the loss of quality of life associated with vision and teeth. Yet the relevant services are also largely uninsured.

**Table 2:** Patient out-of-pocket payments 2005/06

	% of cost
Hospital	2.2

Medical	11.3

Dental	67.9

Medicines	45.9

Aids/Appliances	74.1

Total	17.7

Source: [3]

The apparent (default) explanation is policy inertia, the failure to review the system; or by an overriding year by year concern with the government budget. There are no data to properly document the suffering this causes.

There is no reason why regular reports should not be made available regarding access to and use of services by groups differing geographically, socially, racially and by disease category. The data exist, but are not published. Data should also exist documenting the needless suffering which results from the exclusion of services. Explicit comparisons of groups would sensitise the community and possibly force policies that promote equity. It is possible to surmise that it is precisely for this reason that such information is not produced.

#### Quality and Safety

The issue of adverse events in the Australian health system should dominate all others. However it will be closer to the truth to describe it as Australia's best kept secret. In 1995 the 'Quality in Australian Health Care' (QAHCS) study uncovered an appalling iceberg of avoidable adverse events including large scale unnecessary death. Following publication, virtually nothing effective occurred on a scale commensurate with the problem. Ten years later an editorial in the *MJA *reflected upon this as follows:

'Based on QAHCS outcomes 25 patients die each day in our hospitals from preventable adverse events... we have had report after report... we still have no nationally accepted framework for clinical governance to assure the safety and quality of Australian health services... This ongoing vacuum is an indictment of our Health Ministers and organised medicine' [21].

It appears that following publication the Health Minister of the day accepted the report as being methodologically sound and of great importance. However the representatives of organised medicine pronounced it, without evidence, to be incorrect. Following a change in Government shortly thereafter, the new Health Minister accepted this evidence free conclusion. The study was not repeated to determine validity nor was there an urgent review of safety but simply a series of reports. The cumulative effect of these was summed up, somewhat despairingly in 2006, by the persons initially placed in charge of the reform process in the following way:

*'*One might assume that systematic improvements within the health system are either happening or, at least, well advanced. Regrettable, improvements are still patchy. The greatest challenge for all remains how to achieve universal and systematic changes to the health system within a federated system' [22].

In 2009, the 'Report to Support Australia's First (sic) National Primary Health Care Strategy noted that 'there is currently very little information about the quality of care provided in primary health care... the (US based) Commonwealth Fund survey... found (20 percent) of patients reported experiencing a medical, medicinal or laboratory error [23]. By the end of 2009 it was possible for Healy and Dugdale [24] to write 'realisation is dawning that medical errors are common events' - more than one and a half decades after results from QAHCS became available.

The term 'adverse event' is referred to in only four paragraphs of the National Health and Hospitals Reform Commission final report.

The size of the problem which has been ignored is simply astonishing. If the death rates estimated by the QAHCS are correct then the number of Australians unnecessarily dying is approximately equivalent to a jumbo jet crashing every 2 weeks each resulting in the deaths of 350 Australians. Alternatively, it is equivalent to a repetition of the Bali bombing every 4 days. The cumulative unnecessary deaths since the publication of the QAHCS report would exceed the number of Australians killed in World War 1.

We can only speculate on the reasons why Australian authorities have failed in their most fundamental duty of protecting the lives of their citizens. One possibility is that the magnitudes involved are simply too large for people to believe. (Indeed, it may even seem a little shrill to mention such melodramatic facts!) Human beings form clear expectations concerning the way in which the world they experience operates. As John Maynard Keynes famously commented in the last paragraph of his 'General Theory', after the age of 26-30 most find it very difficult to change these patterns. Australians, their politicians and the medical profession have so long regarded the health system as being safe and amongst the best in the world that results from the QAHCS may simply have been dismissed as absurd, ie conflicting too radically with established patterns of belief. The authorities who endorse or bury such research are well beyond the age of 30. The author's personal experience in attempting to publicise this report is consistent with this hypothesis (see Additional file 3). Nevertheless a prudent government would have repeated the study to disconfirm it.

An alternative explanation is that policy makers, like others, are more responsive to sensational media reports than correctly collected evidence. Cynically, they may only be concerned with the lives of Australians that have been identified and politicised by the press. More probably, however, individuals in governments and bureaucracies (like others) are likely to have focussed only upon their defined area of responsibility and our governance structures have not assigned responsibility for this situation to anyone and lack the flexibility to error learn and act decisively, at least in the health sector.

The National Health and Hospitals Reform Commission did acknowledge the existence of adverse events in muted terms and, chillingly, stated that there is a need for 'culture change'. This same phrase was used over a decade earlier and appears to be code for 'postpone the problem for a generation/leave it to the medical profession, to put their house in order - as they failed to do in the 20^th ^Century'.

## Discussion

### Principles for dynamic adaptation

A common feature of recommended reforms has been what might be described as 'static optimality': A series of one-off recommendations are made for the achievement of the optimal health system. The author has contributed to this literature [14]. Many of the principles suggested are, of course, sound (particularly in the latter reference!) In the optimal system there would be a single purchaser of all services for defined populations. The present irrational, geographic, service-based and disease-based boundaries would be eliminated. Many or possibly all of the performance indicators listed in the NHHRC's April Report would be initially adopted [25].

Below I focus upon a dimension of reform which is seldom considered but, in the light of the previous discussion, would appear to be of greatest importance. The focus is a response to repeated failures; the failure to adequately respond to information when it is available - adverse events and, evolving technologies; failure to investigate or address issues of stated importance - inequity; failure to seek out the nature of health system related social objectives; failure to match policy priorities with the magnitude of the problem and failure to invest in the system navigation equipment necessary for planning the future and responding flexibly to error.

Repeating an earlier theme, the economic (and other) history of the 20^th ^Century has been dominated by technology, innovation and uncertainty - elements for which economics has failed to provide either explanation or guidance. This is reflected in the health economics debate over optimal health systems which, apart from innovation of the moment, have largely ignored the implications of these three dominating themes for system reform. While market capitalism self evidently requires regulation and the market model is manifestly unsuitable for the health sector, the history of capitalism in the 20th Century provides one important insight. The market provides a flexible, adaptive and creative mechanism for allocating resources. The experience of the last 100 years, reviewed comprehensively by Beinhocker [26], suggests the following principles:

• Monopolies, however creative initially, have generally evolved into conservative organisations which commonly fail, a point clearly articulated by Prime Minister Kevin Rudd, in the context of homeland security, when he endorsed the view that 'big departments risk becoming less accountable, less agile, less adaptable and more inward looking' [27].

• Corporations which do not 'reinvent themselves' regularly have a limited life time. Most firms fail after a period of initial creativity and success. The US automobile industry is a dramatic example;

• The engine of progress is often small, innovative enterprise with an idea which has not or cannot be implemented by the larger monopoly/corporation. (The most spectacular recent example is Microsoft's takeover from IBM of the market for desktop computing);

• Growing bureaucracy and overemphasis on due processes are often the reasons why larger 'non-reinventing' corporations loose the innovative advantage (a generalisation again illustrated by the history of Microsoft and IBM); and

• Organisations which have survived, innovated and 'reinvented themselves', have invested heavily in technology and market research - their industrial 'navigation equipment'.

The reform of the Australian health system should be informed by this experience. The reform process should be driven to a significant extent, by the need to achieve dynamic adaptability through time and, in particular, by error learning. None of the reform proposals of which the author is aware, including those of the NHHRC, have emphasised this need. The 'buzz words' are scattered liberally throughout rhetorical passages, but proposals do not show how these translate into policy. Uniquely, the advantages of dynamic adaptability are implicit in the Scotton-Enthoven proposals for Managed Competition although, as elsewhere, Scotton emphasises the static properties of the model [28,29].

The experience summarised above suggests that the following principles should be considered in the reconstruction of the health system, in addition to the principles for static optimality.

1. No part of the Australian health system including the funding of research should be subject to monopoly control. The simplest way of achieving this is to base an integrated health system upon a sub-national unit, either the state health regions or, possibly, fund-holding unit as envisaged in the model of Managed Competition. These units should have a significant degree of autonomy in the way in which they allocate resources. As in the market, diversity maximises the chance of successful innovation and improvement.

The counterargument that differences imply inequity is simply hypocritical. There has been no sustained concern with equity and, as evidenced by their support for PHI, Australians are not particularly interested in the reality of equality. More substantively, when successful elements of a sub-national health system are identified by a national authority they can be mandated for the other health systems. It is doubtful that many would openly defend the structural pretence of equity for the reality of better health especially as both goals can be currently improved.

In this context Canada's leading health economist Bob Evans comments that:

'A particularly interesting feature of the Spanish (experience) is the way in which devolution of political authority to sub-national governments served - against conventional wisdom - to open a democratic window, advancing and securing the universal system in the face of ambivalence (at best) at the national level. (Canada provides a similar example)' [30].

2. Innovation should be 'ongoing' not simply the 'dab innovation' which characterises the present management, but should involve significant and sustained experimentation. As in every other industry this costs money. The expectation that the Coordinated Care Trials commenced in 1995 - by Australian standards, more a 'splash' than a 'dab' - would achieve rapid cost saving without the outlay of significant expenditures was as naive as the expectation by a manufacturer that a new and profitable mode of production might evolve without any venture capital.

3. Innovation should be informed by the careful observation of success overseas, something which has seldom occurred in Australia (the chief exception being the imitation of elements of Canadian Medicare by Medibank in 1994). Despite evidence for over 30 years that the US Kaiser Permanente Corporation has operated highly successful, cost effective, integrated clinics and more recent evidence from the reform of the Veterans Health Service there has been no attempt to seriously study or experiment with their experience. New Zealand's innovations have likewise been ignored. The inward looking nature of Australian policy is epitomised by the failure of the PBAC to look at the international market price of drugs when negotiating with pharmaceutical companies and this has resulted in examples of extraordinary over payment [31]. This is as irrational as a corporation negotiating in a market in terms of the data provided to it by an interested party and ignoring the known prices elsewhere in the market.

4. The above principles cannot be implemented without investment in 'industry navigation equipment'. It is likely that no other industry in Australia spends as little, proportionately, on the marketing, delivery and adaption of their product to customer (social) needs as occurs in the health sector despite the fact that the industry is almost certainly the most complex and important for future wellbeing. The fact that this appears to be true in other countries does not lessen the consequences of this. There has been a failure to invest in health services research on anything much more than a symbolic level and then without serious strategy or plan. While having one of the best data systems in the world, it is largely unused in terms of its real potential for management and evaluation. Research is largely conducted on an ad hoc, or 'on demand' basis, by health departments for specific purposes and commonly results in confidential reports. There is a dearth of creative ideas flowing through to the level of creative planning.

The last serious proposal for comprehensive, coherent reform - the Scotton plan - died at least in part because of the failure to create a new generation of health economists capable of developing such or similar plans and carrying out the prerequisite technical analysis as has been ongoing in the USA, the Netherlands and elsewhere. Perhaps with the wisdom of hindsight it is likely that the existence of serious navigation equipment in the field of new technology would have suggested such extreme uncertainty with respect to workforce requirements that a much greater emphasis would have been given to the training of a flexible workforce capable of varying its level of performance in accordance with the emergence of new technology driven needs.

5. Since the introduction of Medicare in 1994 there has been bipartisan political support for the disregard of serious and well publicised deficiencies in the governance of the health sector as well as those discussed above (Additional file 4). The hypothesis which appears best able to explain this is that at the government level an increasingly important principle is to not create problems for government through reforms which will result in organised opposition from powerful interest groups and only benefit those (the public) who are largely unaware of the benefits that they might receive. If this hypothesis is correct and likely to characterise future political motivation, then an important governance principle is to establish a long term structure which is one stage removed from government with government only responsible for broad policy and funding. The most concerted but largely unsuccessful attempt to significantly improve coordination within the present system was made by the Hospital and Health Services Commission, HHSC, (1973-1976) along with a number of other suggested innovations. Significantly, as discussed later, this group was statutorily independent from government.

### Governance Principles

One example of the allocation of responsibilities based upon (but differing in emphasis from) a modified Scotton model which satisfies many of these requirements is summarised below along with a broad implementation timetable [28,29]. (It is contrasted with Medicare Select later.)

#### De-politicising health

Semi-autonomous commissions should be established at either the State or regional levels responsible for the purchase of all hospital and ambulatory including dental and ophthalmological services. The commission should be directed by a board including representatives of the Commonwealth, State and major providers of services and be able to innovate in both the form of purchasing and the physical organisation of delivery.

#### The reform process

This should be driven by an independent commission which might itself evolve into a statutorily independent body, analogous to the Reserve Bank, for the permanent over-viewing of the health system. The body should not be dominated by 'insiders': health professionals, members of the government health bureaucracy or persons associated with government. (It is too easy for a culture to develop in which common but contestable assumptions become universally accepted. Paraphrasing Donald Rumsfeld, in the Health Sector 'stuff happens' (*cf *adverse events); needed change will be implemented by the responsible department and so on.) The Commission's charter, but not its operation, should, of course, be determined politically (see BCA submission to the NHHRC [32]). Like the Reserve Bank it should have very significant research capacity, for example, incorporating an institute as described below.

#### Regulation and monitoring

The Commonwealth should mandate a minimum package of services and monitor access to these services with penalties for violation of the principles. However there should be capacity for difference and experimentation.

#### Funding

Pooled government revenues should be based upon a predetermined formula with shares unrelated to any element of delivery. The needs adjusted per capita allocation to the purchasing authority, determined by the Commonwealth, should be phased in to replace the status quo. The formula determining the government shares of the funding is irrelevant for system performance.

#### Service provision

Initially, as at present, States should run State hospitals and the private sector run medical, dental, other ambulatory and pharmaceutical services. The Commonwealth should be responsible for negotiating certain prices such as pharmaceuticals and the rebate for fee for service medical services because of the lack of market power by sub-national units.

#### Private Health Insurance

This should initially be unchanged except for the removal of the surcharge and phasing out of life time tables (efficiency measures). The subsidy should initially be maintained. However research is needed to determine the true effect of PHI on the availability of health services to Medicare patients and the size of the subsidy should be determined in the light of this and further research into population preferences with respect to a multi-tier health system.

### Navigation Equipment

At least one, and on the principle of non-monopoly, preferably more statutorily independent institutes should be created similar to but independent from the AIHW. They should have the following functions:

#### Quality monitoring and assurance

The institute should have powers to acquire and require information and to conduct onsite investigation. It should be required to carry out ongoing international collation of techniques for quality assurance and to translate these techniques into a form compatible with the Australian system.

#### Information diffusion

Comprehensive data provision on issues of access and equity, including hospital specific queues by procedure should be made available on the web.

#### Technology and innovation

The institute should proactively seek out new technologies (preferably in conjunction with the relevant medical colleges) and refer them to the relevant technology assessment group for possible inclusion in Medicare.

Results of routine health services research and statutorily determined information should be regularly provided to the public regarding the level of disaggregated service use and the (standardised) quality of different provider groups.

#### Ideas for innovation

The institute should monitor 'good ideas' which have succeeded in other countries and proactively provide these to appropriate bodies throughout the health system.

#### Ad hoc research

It is highly desirable that issues of current public or political interest should be satisfactorily researched and not be the subject of disinformation and unsubstantiated spin. The institute should have a capacity to conduct research on the request of a Minister and on its own initiative. It should have the task of proactively providing relevant information to members of the media who are perceived to be providing factually incorrect information to the public.

#### Blue sky research

There should be a capacity to either conduct or promote additional research of a more long term or exploratory nature.

#### HSR workforce

The institute should be responsible for monitoring and recommending measures to ensure a satisfactory health service research workforce. With current 'dab' funding of HSR there is no career path for health economics and unsurprisingly an almost complete dearth of research into health systems. Part of an institute's function should be the training of such a workforce via cadetships in its research divisions in conjunction with relevant university and government departments.

#### Data

The institute should have a statutory right to all relevant administrative and other data collected by the AIHW but also data which is not collected by the AIHW such as that monopolised by the current Commonwealth Department of Health and Ageing.

In 1973 the Whitlam government created an institute similar to the one described here. The Hospital and Health Services Commission (H&HSC) was statutorily independent and had the ability to analyse, plan, publish, introduce research and recommend policy. It initiated a data based approach to policy development which resulted in the establishment of the Community Health Program which included programs for family medicine, hospital development and initiatives with respect to diagnostic and rehabilitation services, Aboriginal and rural health, health transportation, Aboriginal and rural health and the health workforce [33]. It differed from the present proposal in two important respects. First, it relied upon existing institutions to obtain data. Secondly, it was largely concerned with the formulation and recommendation of policy.

These differences may have proved lethal for its longevity as the Commission was disbanded with the change of government in November 1975 [34]. It is for this reason that the present suggestion is for an institute which cannot be seen as political through its advocacy of policies which may become or already have become politically aligned. Since Australia spends about $100,000 million on health services there is, however, a strong case for (at least) two institutes: one concerned with information and its dissemination and one with options formulation and advocacy along the lines of the H&HSC.

### Timetable

With the exception of the creation of one or more institutes for health services research and evaluation most of the suggested functions, or variants of them, could be achieved relatively quickly as they involve governance and financial flows rather than the creation of new physical infrastructure. Initially the public would feel little effect.

Subsequently, the form of delivery might change substantially as the area based commissions responsible for purchasing services experimented with new methods of delivery (such as Kaiser-like clinics for integrated care). An additional option would be a movement towards Managed Competition as private health funds or other groups negotiated a 'carve out' for a voluntary group. Such a task would involve research into the determination of risk related premiums and likely effects upon cost and equity. (Recent evidence from the Netherland's experiment with Managed Competition suggests that this option might result in inequalities unacceptable even to the unegalitarian Australian public.) Evans [30] notes that from 1995 to 2002 fund specific extra premiums (above needs-based capitation payments) rose from 3 percent to over 50 percent suggesting significant quality differences between schemes. This again illustrates the need for reform based upon careful research and modelling of the impact of different regulatory structures. An indicative timetable is reproduced in Additional file 5.

### The National Health and Hospitals Reform Commission (NHHRC)

In 2009 several major reports were presented to the government. First, a report to Support Australia's First National Primary Health Care Policy conducted a detailed review of issues and options for the reform of Primary Health Care (PHC). The focus of this and the accompanying draft report was primary services delivered by GPs, nurses, allied health providers, Aboriginal health practitioners and pharmacists. Consistent with the problems identified in this paper the key elements included access, coordination, safety and information [23,35].

Next, in an impressive, evidence based report the National Preventative Health Taskforce released its strategy document recommending policies which would, without doubt alter the unhealthy trajectory of Australian society [36]. It is almost certainly correct in its assessment that these policies could prevent 'hundreds of thousands of Australians dying prematurely or falling ill and suffering between now and 2020'. But the barriers to these policies are even larger than those which have blocked serious health sector reform, *albeit *being of the same political origin.

In the context of health sector reform, the most significant document was the final report of the National Health and Hospital Reform Commission (NHHRC) 'A Healthier Future for all Australians' [37]. The report is structurally elegant. It focuses upon health and its achievement in a social context as distinct from the services and financial flows which dominate most proposals for system reform. The report is structured according to important and pivotal principles - connecting care, care at different life stages, rural, mental health etc, inequalities and quality. However, none of the resulting sub-principles, excellent though many of them may be, are likely to become embedded in the system unless the governance structure determining regulation and incentives is appropriate and in this respect the report is deeply disappointing. Its discussion of governance is almost completely absent. In one of the second round submissions Andrew Podger notes that:

'Governance is important. It is not a separate issue from practical measures aimed to improve service delivery and health outcomes. It is the means by which the Australian community can be sure that the health system is delivering what it is there for. Moreover, current governance arrangements are contributing directly to current weaknesses in the quality, effectiveness and efficiency of the Australian health system.' [38]

The Commission's response was to use this quotation to head Chapter 6 and then provide less argument for their recommendations with respect to governance than in the interim report where it represented one page for each of three options under review.

The focus of the Commission's report also has no overlap with the areas of concern discussed in this paper. This is unsurprising. The present paper highlights errors and failures and then sketches a governance system based upon the principle of error learning. The NHHRC, while noting most of the relevant issues somewhere, does not seriously analyse past failures. It never considers the question why major problems and reforms, known and needed for decades, have been ignored and what structural changes are necessary to guard against a perpetuation of this problem.

As with all other proposals Australians would depend upon the wisdom and benevolence of monopoly bureaucrats for the monitoring and implementation of reform; that is error learning and dynamic adaptation would depend upon the same flawed mechanism as at present, namely a department largely driven by short term ministerial concerns and with significant monopoly control over information, research, venture capital and with powerful personal and institutional incentives for the suppression of the information which drives reform, namely information concerning failures and errors.

This casts some doubt upon the wisdom of the structure of the report. Because 'connecting care', care at different life stages, etc are important, it does not follow that they should have determined the structure of the report. Analogously, while the quality of life, not radiotherapy, may be the endpoint of cancer services, a report into these services should not necessarily make quality of life the structural focus if the core problem is an absence of radiotherapy services. The report should focus upon the cause, not consequences of the problem. The integrity of the report depends upon a correct diagnosis of the core problem and not a description, however accurate, of the downstream problems arising from them.

The NHHRC documents so many - probably correct - downstream problems or potential improvements and so many elements of the current system are acknowledged as sub-optimal that perspective on the problems is easily lost; and it appears to be lost in the report. For example, in the draft report, the Quality of Australian Health Care Study (QAHCS), discussed earlier, is viewed as evidence that 'admission to hospital is not without risk' (p 133). This is analogous to acknowledging that 'the Sahara Desert has dry bits'. An alternative perspective is that the QAHCS revealed the greatest non-military, avoidable calamity in Australia's history and that in another context - say, product safety or occupational health and safety - the subsequent disregard of evidence of widespread death and injury would have led to criminal prosecution. But the potential lessons of this astonishing episode of Australia's health history are lost in a sea of largely unfocused detail. In the final report it is simply noted that adverse events can 'cause harm to a person receiving health care... such events cause patients distress and suffering, (and) compromise operational efficiency...' (p 55).

The chief lesson which should have been learned from the QAHCS and the other failures documented here is that government answerable departments - increasingly dedicated to the short term ministerial task of appeasing politically effective groups - are capable of major failures and are increasingly questionable bodies for the short, or even medium term, direction of the health system. There is a need for the separation of policy and system monitoring on the one hand, from implementation and innovation on the other.

The role of government, more generally, has been the single largest theme in the discipline of economics since Adam Smith highlighted its potential shortcomings, and the largest single theme in the economic health policy debate since the introduction of the UK NHS. However there is no echo of this in the NHHRC report. Australian government and governance are implicitly unproblematical, dynamic and wise. The problems discussed by Donald Horne in the 'Lucky Country' have dissolved with time [39].

In the governance structure outlined earlier in this paper the answer to the question 'who guards the guardian' is firstly the Commonwealth regulatory bodies and, secondly, a statutorily independent body which, like the Reserve Bank, has supervisory and regulatory power but no direct responsibility for service delivery. In the Commission's governance structure there is no guardian of the Commonwealth authorities except the Minister and Parliament and it is their impotence on large scale reform which has been the chief problem to date.

The needed governance structure should pre-commit government to certain courses of action and ease the political pain of desirable policy. Analogously, the Reserve Bank may increase interest rates despite short term popular opposition.

These considerations suggest that the NHHRC's chief recommendations with respect to governance are seriously wrong. After a transitionary phase of hospital cost sharing (once believed to be inflationary) they would enshrine a Commonwealth monopoly. The depth of analysis supporting this recommendation in the report, however, is simply lamentable. It argues that '... we heard from many consumers and health professionals - a desire for one health system' (p 147). But the problem documented in Additional file 4 here is the lack of coordination arising from multiple funding sources for the services available to any one individual - integration of primary health and hospital care, step down facilities, etc. This implies the need for a single fund holder for an individual not a single fund holder for all Australia. The case against diversity and experimentation in PHC is simply asserted, 'Our recommendations for ... a transformed comprehensive primary health care platform ...require one government - the Commonwealth Government - to be responsible -... thus we recommend that the Commonwealth Government assumes full responsibility for Primary Health Care Services' (p 148). The argument for economies of scale is untrue and the difficulty tracking border crossing overlooks developments in data processing technology in the last few decades. The Commission argues against regional health authorities because 'there are dangers of 'balkonising' health services, with people's access to care determined by the region they live in' (p 154). The Commissioners do not use irony elsewhere in the report.

In the context of these proposals there is no discussion of regulatory or system incentives for innovation. Almost as an afterthought, however, the Commission does appear to recognise some of the problems inherent in a monopoly and recommends 'Medicare Select' as a longer term governance model. This is a less developed version of Managed Competition than the model advocated by Scotton [40]. It represents an almost complete negation of the arguments for a monopoly. Concern with 'cost and bureaucracy' dissolve and 'innovative approaches to funding' are advocated (p 158). These must equate with employee contributions (one of the most poisonous elements of the US system) or private contributions favouring the wealthy and copayments (disadvantaging the poor). The concerns discussed above are not mentioned.

As 'the devil is often in the detail' Scotton never envisaged his suggestions as being an implementation plan or one which could be fully evaluated on the basis of his broad description. There is no devil in the NHHRC option as there is no detail for it to be in. There is no possibility of Medicare Select being endorsed, as proposed, and its only contribution to the report is that it allows the Commission to encourage the government to reconsider governance.

## Conclusion

Andrew Podger reports that in his capacity of Secretary to the Health Minister, he would suggest a major review of the health system 'almost every other year'. He (the Minister) would respond that articulating clearly the long term direction was as dangerous as "'big bang" reform' [41].

The goal of evidence based policy at the system level appears as far away today as evidence based medicine in the 19^th ^Century and the quotation above indicates a fundamental reason for this. It suggests an obvious conclusion. While elements of the ideal health system are necessarily political - for example the right of people to buy better access to better quality care - measures should be taken which de-politicise reform or at least reduce the political cost of it. The suggestion here is that this be achieved by the dissemination of accurate information about current performance and future options and by the implementation and regulation of policy by de-politicised bodies.

The relative self satisfaction with the Australian system largely arises from a widespread ignorance of the issues discussed here and possibly from crediting the good health of Australians to the institutional and governance arrangements which emerged historically as a result of self-interest, pragmatism and genuine idealism and which are now described, misleadingly, as 'a system'. The existence of similar problems in other countries does not represent evidence of Australia's success but, more probably, of similar histories of defective governance. Bad ideas and bad practices have also been globalised and health system planning and reform has commonly been delegated to interested parties, namely the medical profession, which is generally untrained in system science, and public health bureaucracies steeped in the immovable imperatives of the status quo. However the good health of most Australians cannot justify system satisfaction as its cause is not well understood (deficient research) and as suggested earlier, is probably more attributable to the widespread use of a limited number of effective therapies and effective public health measures than to the (dis)organisation of curative services.

By design and not by default, reform has been incremental where the increments have been tiny, timid and sometimes backwards (eg PHI legislation). While it is true that we are, in large part, prisoners of history this perspective can be overstated and the consequences of this rationalisation of inertia extremely deleterious to the wellbeing of the population. There can be little doubt that major problems with the system have simply been ignored and that this is not attributable to history but to a lack of dynamism, itself attributable in large part to the monopoly control over serious system reform by a politicised bureaucracy.

It has been argued here that the approaches to reform implemented and discussed in Australia have largely missed these dimensions of the problem. Proposals are 'static'. Once achieved the health system will be optimal. Wise, centralised bodies will identify and implement needed change without the need for too much evidence or ongoing experimentation. This approach is reflected in the NHHRC report [25]. Recommendations are listed but they arise from nowhere and in terms of implementation strategies lead nowhere. It is implicitly assumed that with the slightly clarified governance structure, which is recommended, we should trust the bureaucracy to achieve these targets. No broader thinking is revealed in the report. But historically the bureaucracy has comprehensively failed to reform important elements of Australia's health system or even to ensure that the health system is safe. More generally the NHHRC failed to recognise any of the key themes discussed here and, in contrast, recommended the reinforcement of the single most harmful element in the system, the monopolisation of the entire country's health system by a single body. Belief in the flexibility, benevolence and wisdom of monopolies in either the public or private sectors simply misses the major lesson of the 20^th ^Century.

In view of the overwhelming evidence from outside the health system that progress depends upon the 'reinvention' of organisations from time to time, the final conclusion here is that the health sector, which represents the most expensive industry in the country - 10 percent of the national use of its resources - and a major potential source of future wellbeing, requires a comprehensive, long term and long overdue review of all aspects of its operation. This should not be based solely upon the collation of opinions, however authoritative, and the balancing of interest groups. It should include a detailed and published analysis of the system's strengths, weaknesses and opportunities

## Competing interests

The author declares that they have no competing interests.

## Authors' contributions

JRJR is the sole author of this work.

## Supplementary Material

Additional file 1 The ungenerous country. Click here for file

Additional file 2 Measuring equity: The author's experience. Click here for file

Additional file 3 Ideas or authority: response to the QAHCS. Click here for file

Additional file 4 The continuity of reform paralysis. Click here for file

Additional file 5 **Timetable to a (yet unproven) Managed Competition (NHHRC Option 3) **[43-48]. Click here for file

## References

[B1] Richardson J Sinha K Iezzi A Why we should not minimise Cost per QALY: Theoretical and Empirical Evidence, Research Paper 38 2009 Melbourne: Centre for Health Economics, Monash University

[B2] Anderson M Richardson J McKie J Iezzi A Queues, Private Health Insurance and Medicare: Is Economics the Driving Force? Research Paper 23 2007 Melbourne Centre for Health Economics, Monash University

[B3] Australian Institute for Health and Welfare Health Expenditure Australia 2007-08 2009 Canberra: AIHW

[B4] Easterlin RA Income and happiness: Towards a unified theory The Economic Journal 2001 111 465 484 10.1111/1468-0297.00646

[B5] Frey B Stutzer A What can economists lean from happiness research? Journal of Economic Literature 2002 XL 402 435 10.1257/002205102320161320

[B6] Australian Government Intergenerational Report 2007 Budget Papers, Department of Treasurey 2007 Canberra ACT Australia

[B7] Productivity Commission Economic Implications of an Ageing Australia: Productivity Commission Draft Research Report 2004 Canberra: Australian Government

[B8] National Health and Hospitals Reform Commission (NHHRC) A Healthier Future for all Australians - Interim Report 2009 http://www.nhhrc.org.au 10.5694/j.1326-5377.2009.tb02845.x 19807629

[B9] Richardson J Robertson I Ageing and the cost of health services Policy Implications of the Ageing of Australia's Population 1999 Melbourne: Productivity Commission and Melbourne Institute of Applied Economics and Social Research

[B10] Richardson J McKie J Sinha K A Critique of Efficiency Focussed Economic Evaluation in the Context of a NHS: The case for Empirical Ethics, Research Paper 34 2009 Melbourne: Centre for Health Economics, Monash University

[B11] Diener E Suh E Lucas R Smith H Subjective well-being: Three decades of progress Psychological Bulletin 1999 125 276 302 10.1037/0033-2909.125.2.276

[B12] Duckett S Jackson T The new health insurance rebate: an inefficient way of assisting public hospitals Medical Journal of Australia 2000 172 439 442 10870538 10.5694/j.1326-5377.2000.tb124043.x

[B13] Duckett S Private care and public waiting Australian Health Review 2005 29 87 93 15683360 10.1071/ah050087

[B14] Richardson J Priorities of health policy: Cost shifting or population health Australia and New Zealand Health Policy 2005 2 10.1186/1743-8462-2-1 15679895 PMC548139

[B15] Deeble J Le plus ca change: Recollections of a retiring health economics The Chalmers Oration 2009, 23 July 2009 2009 Flinders University Medical School: Australian Healthcare and Hospitals Association

[B16] Richardson J Deeble J Statistics of Private Medical Services in Australia 1976 1982 Canberra: Australian National University

[B17] Richardson J Mooney G, Scotton R The health care financing debate Economics and Australian Health Policy 1999 St Leonards: Allen & Unwin 192 213

[B18] Robertson I Richardson J The effect of funding upon hospital treatment: The case of coronary angiography and coronary artery revascularisation procedures following acute myocardial infarction Medical Journal of Australia 2000 173 291 295 11061397 10.5694/j.1326-5377.2000.tb125658.x

[B19] Department of Health and Ageing Australian Statistics on Medicines 2007 2009 Canberra: Australian Government

[B20] Reeder CE Nelson AA The differential impact of co-payment on drug use in a Medicaid population Inquiry 1985 22 396 403 2934334

[B21] Weyden M Van Der The Bundaberg Hospital scandal: Th need for reform in Queensland and beyond Medical Journal of Australia 2005 183 284 285 16167864 10.5694/j.1326-5377.2005.tb07054.x

[B22] Baraclough B Birch J Health Care Safety and Quality: Where have we been and where are we going? Medical Journal of Australia 2006 184 S48 S50 16719736 10.5694/j.1326-5377.2006.tb00362.x

[B23] Department of Health and Ageing Primary Health Care Reform in Australia: Report to support Australia's First National Primary Health Care Strategy 2009 Canberra: Australian Government

[B24] Healy J Dugdale P Patient Safety First: Responsive Regulation in Health Care 2009 Sydney: Allen and Unwin

[B25] National Health and Hospitals Reform Commission (NHHRC) Beyond the Blame Game: Accountability and Performance Benchmarks for the next Australian Health Care 2008 http://www.nhhrc.org.au

[B26] Beinhocker E The Origin of Wealth 2006 Boston: Harvard Business School

[B27] The Age Rudd scraps plan for new department, Coastguard The Age 2008 Melbourne: Fairfax

[B28] Scotton R Mooney G, Scotton R Managed competition Economics and Australian Health Policy 1999 Sydney: Allen & Unwin 214 231

[B29] Scotton R Risk-adjusted capitation system and payment framework Productivity Commission Workshop on Managed Competition; 23 August Canberra. AusInfo 2002

[B30] Evans R Fellow travellers on a contested path: Power, purpose and the evolution of European health care systems Journal of Health, Politics, Policy and Law 2002 30 277 293 10.1215/03616878-30-1-2-277 15943397

[B31] Spinks J Richardson J Is Australia paying too much for generic pharmaceuticals Research Paper 44 2009 Melbourne: Centre for Health Economics, Monash University

[B32] Business Council of Australia Fit for the Job: Adapting to Australia's New Health Care Challenges 2009 Melbourne: Business Council of Australia

[B33] Leeder S Lewis MJ Learning from past commissions Medical Journal of Australia 2008 189 268 269 18759724 10.5694/j.1326-5377.2008.tb02023.x

[B34] Southby RF Health care reform: Looking back to go ahead Medical Journal of Australia 2008 189 33 34 18601639 10.5694/j.1326-5377.2008.tb01892.x

[B35] Department of Health and Ageing Building a 21st Century Primary Health Care System, A Draft of Australia's First National Primary Health Care Strategy 2009 Canberra: Australian Government

[B36] National Preventative Health Taskforce Australia: The Healthiest Country by 2020. National Preventative Health Strategy - the roadmap for action 2009 Canberra: Australian Government

[B37] National Health and Hospitals Reform Commission (NHHRC) A Healthier Future for all Australians: Final Report June 2009 http://www.nhhrc.org.au/internet/nhhrc/publishing.nsf/Content/nhhrc-report Accessed August 2009 10.5694/j.1326-5377.2009.tb02845.x 19807629

[B38] Podger A Submsision 292 to the National Health and Hospital Reform Commision Interim Report Second Round Submissions 2009 Canberra Common wealth Dept of Health and Ageing, Ageing CDoHa

[B39] Horne D The Lucky Country: Australia in the Sixties 1964 Melbourne: Penguin Books

[B40] Productivity Commission Managed Competition in Health Care Workshop Proceedings; Canberra. AusInfo 2002

[B41] Podger A A Model Health System for Australia 2006 Inaugral Menzies Health Policy Lecture

[B42] Tiffen R Gittins R How Australia Compares 2004 Cambridge: Cambridge University Press

[B43] Richardson J Reducing the incidence of adverse events: Results from a modified Delphi technique Australia and New Zealand Health Policy 2008 5 10.1186/1743-8462-5-25 19014562 PMC2596159

[B44] National Health Strategy The Australian Jigsaw: Integration of Health Care Delivery, Issues Paper No 1 July 1991 1991 Canberra: Department of Health, Housing and Community Services

[B45] Senate Community Affairs References Committee Healing our Hospitals: Report on Public Hospital Funding 2000 Parliament of Australia

[B46] (The) Allen Consulting Group Governments Working Together: A Better Future for all Australians 2004

[B47] Productivity Commission Australia's Health Workforce, Research Report 2005 Canberra: Productivity Commission

[B48] Richardson J Financing Health Care: Short Run Problems, Long Run Options. Paper presented to the Health Reform Forum, Melbourne Business School 19 September 2002 2003 Melbourne: Centre for Health Economics, Monash University

