# Supplementary material for: Steering without navigation equipment: the lamentable state of Australian health policy reform
Source: Aust New Zealand Health Policy. 2009 Nov 30;6:27. doi: 10.1186/1743-8462-6-27 (PMC2791101; doi:10.1186/1743-8462-6-27)
Supplement: Additional file 1 — The ungenerous country. [file 1743-8462-6-27-S1.DOC]

For those believing that Australians are egalitarian and that the political cliché of a ‘fair go’ and ‘mateship’ describe the Australian character, see Table 1. Casual observation indicates greater ‘downward envy’ in our national press and character than concern with a fair distribution and compassionate treatment of the disadvantaged.
