# Supplementary material for: Steering without navigation equipment: the lamentable state of Australian health policy reform
Source: Aust New Zealand Health Policy. 2009 Nov 30;6:27. doi: 10.1186/1743-8462-6-27 (PMC2791101; doi:10.1186/1743-8462-6-27)
Supplement: Additional file 2 — Measuring equity: The author's experience. [file 1743-8462-6-27-S2.DOC]

Following the studies referred to in the text the author successfully obtained NHMRC funding for a project to evaluate the change in the equity of Medicare through time. This required information from the Commonwealth Department of Health and Ageing (DHA) which has been given monopoly control over historical data relating to the use of medical services. Over a five year period – 2001-2006 – and despite repeated promises that data would be shortly forthcoming, the required information was never provided as a result of a series of ad hoc ‘problems’, delays and ‘higher priorities’ at the DHA. Following the non reply to three of the four letters sent by the Deputy Vice Chancellor, Research, at Monash University the author concluded that these data would never be forthcoming as they were likely to demonstrate the failure of the Department and Government to increase the equity of Medicare. At the time of writing (September 2009) the data request is still with the DHA but there has been no response to the Monash communications for 3 years.

This episode may or may not be unique. However it demonstrates the danger of granting monopoly power over public data to a body with a strong interest in the way in which it is used.
