# Supplementary material for: Steering without navigation equipment: the lamentable state of Australian health policy reform
Source: Aust New Zealand Health Policy. 2009 Nov 30;6:27. doi: 10.1186/1743-8462-6-27 (PMC2791101; doi:10.1186/1743-8462-6-27)
Supplement: Additional file 3 — Ideas or authority: response to the QAHCS. [file 1743-8462-6-27-S3.DOC]

Because of the puzzling lack of policy following the QAHCS, Richardson and McKie conducted a small scale study which involved contacting individuals with a track record in adverse events or experts in hospital and medical safety [43]. This group was asked to nominate policies which might be immediately implemented and to estimate the time before these policies would become effective. Results from the first round of this ‘Delphi-’ research was classified and circulated for comment and for respondents to estimate time lags on policies. The result was a set of 41 policies spanning the health system. Some of the recommendations were common sense. For example, it was suggested that, following a major operation, a hospital should be required to have competent medical staff in situ during the recovery period in case of complications. This requirement did not (does not?) exist. In sum, we carried out the obvious first step of a policy reform agenda, namely the collation of ideas.

The response to this report was telling. Informal feedback challenged our authority to do such work and at least one reviewer wished to know the names of the experts consulted as, presumably, the policies needed to be assessed by the authority of the initiator and not by their own merits.

The author sent the report to the current government twice without reply. When sending it a third time I asked for acknowledgement of its receipt and duly received a terse note thanking me for my interest in adverse events signed, not by a member of the Minister’s staff to whom the letter had been sent, but by an officer in the DHA.

This suggests a culture in which policies are driven by due process and authority and not by urgency.
