# Supplementary material for: Steering without navigation equipment: the lamentable state of Australian health policy reform
Source: Aust New Zealand Health Policy. 2009 Nov 30;6:27. doi: 10.1186/1743-8462-6-27 (PMC2791101; doi:10.1186/1743-8462-6-27)
Supplement: Additional file 4 — The continuity of reform paralysis. [file 1743-8462-6-27-S4.DOC]

1975 The Commission concluded that health... services should be more closely integrated in the future to provide appropriate care and support. (Australian Government Social Welfare Commission) [44]

1976 There is an immediate need to rationalise the mosaic of health and welfare services which is marked by duplication of roles and responsibilities. (Coombs Inquiry) [44]

1977 We have found evidence of a degree of overlap duplication, proliferation and excessive administration with regard to Commonwealth (health and welfare) programs. (Bailey Review) [44]

1980 The present arrangements are fragmented and tend to work against effective integration and utilisation of the various health services. (Jamieson Inquiry into the Efficiency of Administration of Hospitals) [44]

1988 There is clear consensus amongst states and territories that existing programs providing related services... are separate, distinct and often uncoordinated. (National Community Consultation for Review of the Home and Community Care Program 1988) [44]

1990 Provision of funding through separate budgetary allocations or by different levels... the optimum use of resources. (Senate Select Committee on Health and Health Insurance 1990) [44]

1991 The separation of funding of non-inpatient specialist, GP and diagnostic services... has created the strong incentive for cost-shifting, rather than a central focus on overall efficiency and optimum health outcomes... Programs... lack cohesion. (National Health Strategy, 1991 The Australian Health Jigsaw: Integration of Health Care Delivery, Issues Paper No 1) [44]

2000 The Committee... believes that the lack of a national health policy reflects the fragmented nature of the health system. (Senate Community Affairs References Committee, Healing our Hospitals: Report on Public Hospital Funding, December 2000) [45]

2004 In many cases the way the two levels of government interact does little for the quality of services received by the community, due to duplication, inefficiency and lack of coordination. (The Allen Consulting Group 2004) [46]

2006 There are fragmented roles, responsibilities and regulatory arrangements... inadequate coordination between governments, planning, education and service providers... inflexible regulatory practices... perverse funding and payments incentives... and entrenched custom and practice. (Productivity Commission 2006) [47]

2008 No level of government has a detailed understanding of all aspects of the health sector... there is widespread dissatisfaction with the fragmentation of services and difficulties with navigating a complex system... a strong message we heard... was a desire for ‘one health system’. (National Health and Hospitals Reform Commission 2009) [8]

2009 Service delivery is characterised by multiple and fragmented funding streams and service delivery arrangements can be inflexible and poorly coordinated, both within primary health care but also across hospitals, aged care and specialist care. (Building a 21^st^ Century Primary Health Care System, Draft of the First National PHC Strategy, Department of Health and Ageing, 2009) [35]
