# Supplementary material for: Steering without navigation equipment: the lamentable state of Australian health policy reform
Source: Aust New Zealand Health Policy. 2009 Nov 30;6:27. doi: 10.1186/1743-8462-6-27 (PMC2791101; doi:10.1186/1743-8462-6-27)
Supplement: Additional file 5 — Timetable to a (yet unproven) Managed Competition (NHHRC Option 3) [43-48]. [file 1743-8462-6-27-S5.DOC]

| t_0_: Methodology and Cost calculations | Enquiry: what to pool. Regulation  Expected spending  Research into pooling methodology |
| --- | --- |
| t_1_: Pooling | 1. Regional authorities (n = 15) receive a single budget equal to present region spending 2. Initially 100 percent reimbursement of overspending 3. Reimbursement of providers as occurs presently 4. HIC a possible agent 5. Public hospital reimbursement by DRG 6. Evaluation of administrative and financial effects |
| t_2_: Early Transition 1 | 1. Regional budgets adjusted 5 percent per annum towards ‘expected value’ determined by a risk population based framework 2. Regions permitted to alter specified relationships (eg limited preferred provider contracts but preservation of ‘default payments’; employment of allied health personnel; introduction of integrated information, QA systems) 3. Enquiry: problems arising; role of PHI |
| t_3_: Late Transition 1 | 1. Regional budgets set equal to the ‘expected budget’ 2. Flexibility and discretion increased eg no or low default payment for non-contract providers; elimination of high risk (low quality) hospital departments; construction of (Kaiser style) vertically integrated clinics. Possible integration with aged and some welfare services 3. Assessment of transition, governance and outcome effectiveness of regulation 4. Enquiry into feasibility and benefits of private ‘carve outs’ |
| t_4_ Transition 2 | 1. Private sector ‘carve outs’: transfer of full budgets per person to registered accredited groups, regulated as in the Scotton proposal 2. Phase out of current PHI 3. Ongoing review of performance |
